# Preparation of Gallic Acid-Grafted Chitosan Using Recombinant Bacterial Laccase and Its Application in Chilled Meat Preservation

**DOI:** 10.3389/fmicb.2018.01729

**Published:** 2018-08-03

**Authors:** Meixia Zheng, Chong Zhang, Ying Zhou, Zhaoxin Lu, Haizen Zhao, Xiaomei Bie, Fengxia Lu

**Affiliations:** Laboratory of Enzyme Engineering, College of Food Science and Technology, Nanjing Agricultural University, Nanjing, China

**Keywords:** bacterial laccase, chitosan, gallic acid, grafting, chilled meat preservation

## Abstract

To improve the antibacterial and antioxidant properties of chitosan (CS), CS grafted with gallic acid (GA) using recombinant bacterial laccase from *Bacillus vallismortis* fmb-103 (fmb-rL103) as a catalyst. The structures of grafted chitosans were identified using Fourier transform infrared spectroscopy (FT-IR) and UV visible spectrum (UV–Vis spectroscopy). After gallic acid grafting, the antibacterial properties of chitosans against *Pseudomonas*, *Acinetobacter*, *Brochothrix thermosphacta*, *Escherichia coli*, *Staphylococcus aureus*, *Salmonella*, and *Listeria monocytogenes* were significantly improved. Meanwhile, 1,1-Diphenyl-2-picrylhydrazyl (DPPH) radical scavenging results showed that the antioxidant properties of grafted CS increased as well. The preservative effects of the grafted chitosan on chilled meat were then investigated. For this purpose, the quality indexes of the chilled meat during the storage were monitored, including total bacterial count, total basic volatile nitrogen (TVB-N) content, pH value, color and thiobarbituric acid reactive substances (TBARS) and so on. The results showed that coating with the grafted chitosan retarded the growth of spoilage bacteria, and decreased TVB-N and TBARS values of meat. The shelf life of chilled meat coated by CS grafted with GA (GA-g-CS) also extended from 6 days to 18 days at 4°C. These results provided a theoretical basis for the future application of the GA-g-CS in the preservation of chilled meat.

**Highlights:**

(1) The temperature and pH-stable bacterial laccase was used to synthesize gallic acid grafted chitosan.
(2) Antioxidant and antibacterial properties of chitosan were improved through grafting gallic acid.
(3) Storage properties of chilled meat were improved by coating with gallic acid grafted chitosan.

## Introduction

Chitosan (CS) is a good substance for food preservation due to its non-toxic, biodegradable, biocompatible and antimicrobial features. It derives from the alkaline de-acetylation of chitin, which is extracted from fungal species or from the sea creatures’ exoskeleton such as prawns crayfish, lobster, crab and shrimp (Shukla et al., 2013). The antioxidant and antibacterial activities of CS can be improved after grafting with small molecules. The structure of chitosan contains two active groups: hydroxyl group (at C6 carbons) and amino group (at C3). Electrostatic interactions with other compounds can be established if these functional groups protonated (Liu et al., 2012).

Natural phenolic, such as chlorogenic acid, ferulic acid, caffeic acid, and gallic acid, are good candinates to be grafted with CS backbones (Cirillo et al., 2016). Up to now, there were four types of grafting techniques, such as free radical mediated grafting, carbodiimide based coupling, enzyme-catalyzed grafting and electrochemical methods, have been developed to graft phenolic groups onto CS (Liu N. et al., 2018). In previous studies, Liu J. et al., 2018. synthesized caffeic acid (CA) –grafting-CS (CA-g-CS) in hydroxyl peroxide redox and ascorbic acid system (Liu J. et al., 2018). Fan et al. (2017) grafted CS with chlorogenic acid through free-radical-induced protocols. Xie et al. (2014) reported that CS can be grafted with gallic acid in the presence of carbodiimide and hydroxybenzotriazole. In addition, Gray et al. (2011) grafted CS with caffeic acid through electrochemical methods. Yang et al. (2016) modified chitosan by cinnamic acid grafting using fungal laccase. Among the aforementioned ways to modify CS, enzymatic grafting method with phenolic acid has some advantages over the other three methods. Firstly, enzymes commonly used for the synthesis of phenolic acid-g-CS are much cheaper than carbodiimide. Secondly, immobilized enzymes can be used repeatedly (Vittorio et al., 2016). Thirdly, deprotection and full protection step involved in chemical coupling reaction can be eliminated, for the high selectivity and specificity of enzymes. Moreover, enzyme-catalyzed grafting method is more eco-friendly and much safer than chemical coupling method (Aljawish et al., 2014).

Laccase is a widely used catalyst for the phenolic acid grafting to CS. Laccases (benzenediol: oxygen oxidoreductases; EC 1.10.3.2) are classified as polyglycovine oxidase that generate free radicals and utilize molecular oxygen as an electron acceptor (Fokina et al., 2016). They are capable to oxidize a large variety of aromatic substrates, while oxygen is reduced to water as the sole by-product (Jahangiri et al., 2018). According to the origin, laccases can be divided into plant laccases, fungal laccases and bacteria laccases (Yadav et al., 2018). Bacterial laccases have advantages over fungal counterpart, such as thermal-stable (Berini et al., 2018) and pH-tolerant (Ece et al., 2017). Furthermore, bacterial laccases do not need glycosylation and can be over expressed in *Escherichia coli* within a short fermentation period.

Meat spoilage was steadily concerned over by meat industry and retail market, since it causes substantial losses of up to 40% of production (Lahmar et al., 2018). The main cause of meat corruption is the growth of microbes including spoilage and pathogenic bacteria. Microbial breeding can result in large losses of meat quality and potential safety hazard. Pork represents a common animal protein source. It was estimated that the world’s per capita consumption was 12.43 kg (Custódio et al., 2018). Therefore, properly managing and handling pork preservation is a scientific challenge with great economic impact. Many efforts have been made to prolong the shelf life of pork, such as chilling and freezing. Although chilled meat has longer shelf life than ordinary meat, it still can’t meet consumers’ demand. Thus, various natural antioxidants from plant sources had been considered for meat preservation (Kim et al., 2016). However, these natural antioxidants are considerably expensive. An economic and effective material to improve shelf life of meat is urgently needed.

In the study, gallic acid-grafted CS was prepared using recombinant bacterial laccase from *Bacillus vallismortis* (fmb-rL103). The properties and antioxidant and antibacterial activities of resulting GA-g-CS were assayed. GA-g-CS was then applied for chilled meat preservation, for the purpose of enhancing product storage quality.

## Materials and Methods

### Bacterial Laccase and Other Chemicals

Refer to the method of Sun et al. (2017), recombinant bacterial laccase (fmb-rL103) with temperature and pH stability was prepared through heterologous production of laccase from *Bacillus vallismortis* fmb-103 in *E*. *coli*, and purified using an affinity chromatography method. Specifically, crude enzyme was first heated at 75°C in water bath for 10 min, then centrifuged at 8000 rpm and 4°C for 30 min. The supernatant was collected for the further purification by NTA (nickel column, GE product). Imidazole (final concentration of 5 mM) was added to the enzyme sample to enhance the adsorption capacity of column. The column was balanced with imidazole (20 mM) before and after injection. Finally, the samples were eluted with 25 mM imidazole. The aforementioned process was repeated until pure laccase was obtained.

Gallic acid was purchased from Sigma-Aldrich Chemical Co. (St. Louis, MO, United States). Chitosan MMW (Middle molecular weight, 50 kDa) were purchased from Yongsheng Biotechnology (Shanghai, China). Pork (boneless loin, Sushi) was purchased at Suguo market in Nanjing, China. All other conventional reagents were of analytical grade.

### GA-g-CS Preparation

Enzymatic synthesis of GA-g-CS was performed using a heterogeneous grafting method (Liu N. et al., 2018). CS powders (1 g) were dispersed in buffer solution (PBS 45 ml, pH 6.5) and then mixed with 5 mL of GA solution in methanol (100 mM) and 150 μL of fmb-rL103 (1000 U/mL). The mixtures were kept at 40°C for 3 h with continuous stirring (100 rpm/min). Then the solution was cooled to room temperature and pellets were collected by centrifugation (8000×g, 15 min, 4°C). In order to remove the free GA, the collected particles were washed in ethanol and water in turn. Then the products were dried by vacuum freeze-drying. The dried powder was stored at 4°C for the later analysis. All experiments were performed in triplicate.

### UV-Vis and FT-IR Analysis

CS, GA-CS and GA-g-CS powder were dissolved in acetic acid solution (1%, v/v) at a concentration of 1 mg/mL individually. Then, the samples were subjected to full-wavelength scanning from 200 to 600 nm using a UV-visible (UV-Vis) spectrophotometer (UV-2450/2550, Shimadzu, Kyoto, Japan).

The powdered samples were subjected to Fourier transform infrared spectroscopy (FT-IR) (Nicolet iS50, Madison, WI) (128 scans, 10 kHz, 500–4000 cm^−1)^. The ambient gas was air. The grafting situation of GA and CS was determined by bond analysis.

### Antioxidant Activity

Antioxidant activity is assayed through the scavenging activity of 1,1-Diphenyl-2-picrylhydrazyl (DPPH) radical based on the reported method (Xie et al., 2014) with some modifications. CS, GA-CS and GA-g-CS powder were dissolved in acetic acid solution (1%, v/v) at concentrations of 0.5, 0.8, 1, 1.2, and 2 mg/mL, respectively. A methanolic solution (2 mL) containing DPPH radicals (2 mM) was mixed with 2 mL of CS solution. The mixture left to stand in the dark at 33°C for 30 min. The absorbance of the resulting solution (*A*_sample_) was measured at 517 nm. The control used acetic acid solution (1%, v/v) instead of a sample solution. The absorbance of the control is denoted by *A*_control_. The free radical scavenging activity was calculated by the following equation:

$$\text{DPPH scavenging ability}(\%)=(1-A_{\text{sample}}/A_{\text{control}})\times 100\%$$

The EC_50_ value, the antioxidant concentration, reduced half of DPPH radicals.

All experiments were done in triplicate.

### Antibacterial Activity

The antibacterial activity of CS, CS-GA and GA-g-CS against *Staphylococcus aureus* (CMCC 26003), *Escherichia coli* (ATCC 25922), *Pseudomonas* (isolated from pork by the Enzyme engineering laboratory of Nanjing Agricultural University), *Acinetobacter* (isolated from pork by the enzyme Engineering laboratory of Nanjing Agricultural University), *Brochothrix thermosphacta* (isolated from pork by the Enzyme engineering laboratory of Nanjing Agricultural University), *Staphylococcus aureus* (CMCC 26003), *Salmonella acillus subtilis* (CICC 21483), and *Listeria monocytogenes* (CICC 21662) were determined using the Oxford Cup method with some modifications (Li et al., 2018). CS, GA-CS and GA-g-CS (0.1 g) was dissolved in 0.1 M hydrochloric acid solution (100 ml), respectively. The plates containing the indicated culture (0.1 ml; bacterial concentration, 10^5^–10^6^ cfu/mL) were prepared and punched with a puncher. The sample solution (0.2 ml) was added to the hole of the plates. The plates were then incubated in an incubator. The diameters of bacteriostatic ring were determined. All experiments were performed in triplicate.

### Application in the Preservation of Chilled Meat

#### Coating Chilled Meat With GA-g-CS Complexes

The boneless loin was divided into different portions (80 g/portions; 60 portions/ treatment), averagely impregnated in CS, GA-CS, and GA-g-CS solution (pH = 2.99 ± 0.05; dispersing 1 g CS, GA-CS and GA-g-CS powders in 100 ml 1% acetic acid) at 25°C for 1 min and dried. The total amount of chitosan (<0.2 g/kg pork) is far less than the limit value (6 g/kg pork) as a membrane agent according to the National Food Safety Standard - Standards for Uses of Food Additives Chinese Standard (GB 2760-2014) (2014). The treated samples were placed in homogeneous bags, sealed and stored in the refrigerator at 4°C for 20 days. Sampling was conducted every 2 days until the end of storage. All experiments were performed in triplicate.

#### Color

To assess the meat surface color properties (lightness *L*^∗^ and redness *a*^∗^), a Minolta Color Reader (CR-400; Minolta, Japan) was used. It was calibrated with the aid of a white plate before use (*L*^∗^ = 86.3, *a*^∗^ = 0.3165, and *b*^∗^ = 0.3142). All of color measurements were made on five different parts of the meat surface.

#### Assay of pH

The method for determining pH refered to the method of Chinese Standard (GB 5009. 237-2016), 2016b. Boneless loin (10 g) was homogenized (PT-MR2100, Kinematica, Switzerland) in 90 mL distilled water. Then pH values were measured using a digital pH meter (Orion 3-Star Plus, Thermo Scientific, United States) on each sampling day. Triplicate readings were obtained.

#### Evaluation of Thiobarbituric Acid Reactive Substances (TBARS)

Lipid peroxidation was measured using a TBARS assay according to the method described by (Lahmar et al., 2018) with slight modifications. To be exact, each sample (10 g) was placed in 15 mL of mixed solution (0.45 M trichloroacetic acid, 3.4 mM EDTA, 3.4 mM propyl gallate) and homogenized at 14,000 rpm for 1 min. After that, samples were filtered through Whatman No. 1 papers. Filtered samples (5 mL) and 5 mL of 0.02 M 2-thiobarbituric acid (TBA) were added in test tubes, mixed and then incubated at 100°C (water bath) for 40 min. Absorbance at 530 nm (A_530_) was measured using a UV-Vis spectrophotometer (Shimadzu, Kyoto, Japan). The results were expressed as mg malonaldehyde (MDA)/kg of pork. The standard curve was made using TEP solutions with different concentrations.

#### Determination of TVB-N

Meat freshness (TVB-N level) was measured using the Automatic Kjeldahl method according to the Chinese Standard (GB 5009. 228-2016) (2016a). Homogenized pork loin (10 g) was added to 75 ml distilled water and incubated for 30 min. In this period, the blank reagent was measured instrumentally to get the blank value. Afterward, 1 g MgO was added to the distilled pipe containing the processed sample and then immediately connected to the distiller. The determination was conducted according to the set conditions. The TVB-N content was calculated as:

$$\text{X}=\frac{\left({\text{V}}_{1}-{\text{V}}_{2}\times \text{c}\times 14\times 100\right)}{\text{M}}$$

In this equation, X is the content of TVB-N in the sample (mg/100 g), V_1_ is the consumed hydrochloric acid volume for sample titration (mL), V_2_ is the consumed hydrochloric acid volume for blank reagent (mL), c is the concentration of hydrochloric acid solution used for titration (mol/L), m is the weight of sample (g). 100 is the unit conversion factor.

#### Microbiological Analysis

Total bacterial count (APC) and coliform group were determined by plate counting method according to Chinese Standard (GB 47892-2010) (2010). Pork samples (10 g) were blended with distilled water (90 mL), and then serially diluted. One milliliter diluted sample was spread on colony counting plate to determine total bacterial counts (APC). The plates were incubated at 37 ± 1°C for 24 h. *Escherichia coli* was incubated on plates containing eosin methylene blue medium, for 48 h under the same conditions according to Chinese Standard (GB 4789. 3-1994) (1994). Bacterial colonies on the plates were counted and expressed as log_10_ colony-forming units (cfu/g) of meat.

### Statistical Analysis

The data were represented as mean ± standard deviation (SD) of triplicates. Duncan’s multiple range tests and one-way analysis of variance (ANOVA) were performed using SPSS 16.0 software for multiple comparisons. Differences were deemed statistically significant if *p* < 0.05.

## Results and Discussion

### Synthesis of GA-g–CA Complexes Using Recombinant Bacterial Laccase

**FIGURE 1:**
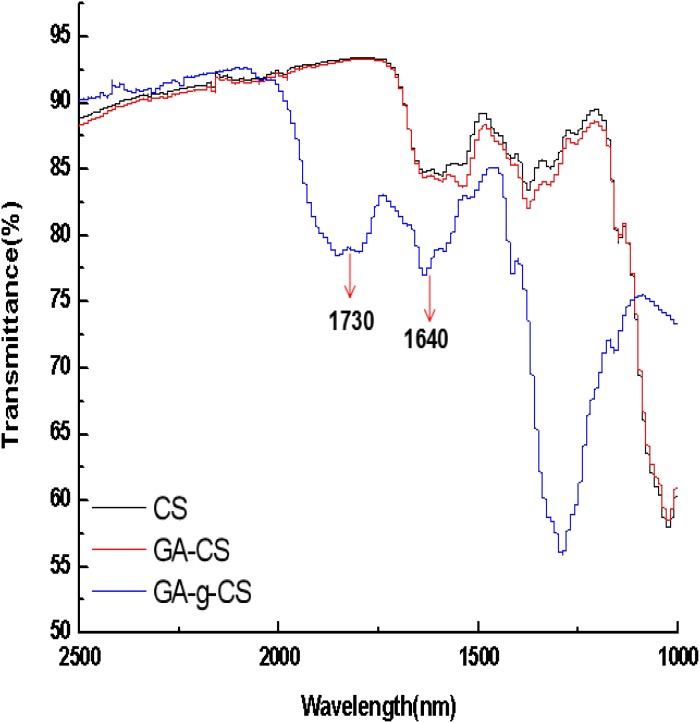
The FTIR spectra of CS, GA-CS and GA-g-CS.

After grafting with GA, CS had a stable brown color. It was speculated that GA was successfully grafted onto CS. FTIR spectroscopy was further performed to validate the grafting process. **Figure 1** shows the FTIR spectra of control CS, GA-CS and GA-g-CS covalent complex. Only the FTIR spectra between 2500 and 1000 cm^−1^ were illustrated, since the FTIR spectra between 4000–2500 cm^−1^ and 1000∼400 cm^−1^ for all the samples were almost identical. As can be seen from **Figure 1**, the FTIR spectrum of GA -CS was similar to that of control CS. GA-g-CS found some changes in the spectra of 1730 and 1640 cm^−1^, which represented C = O stretching in esters and -C = O stretch of chitosan amide (Wei and Gao, 2016). This result indicated ester bonds and amide bonds were formed between GA and CS under the catalysis of fmb-rL103.

**FIGURE 2:**
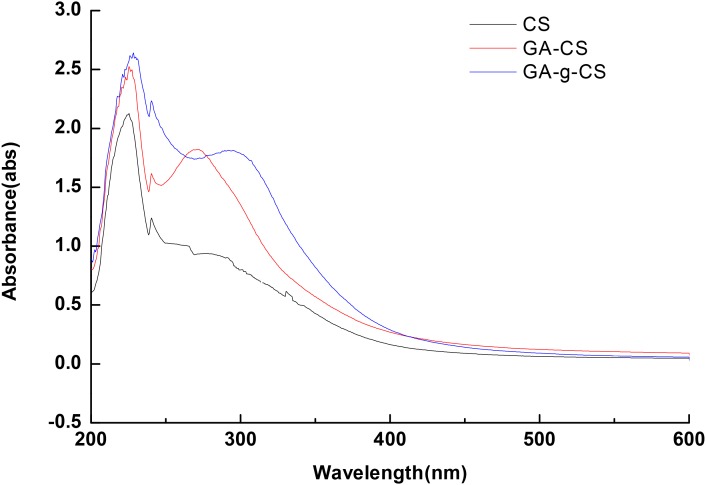
The UV-vis spectra of CS, GA-CS and GA-g-CS.

The formation of covalent bond was further examined by means of UV-vis spectroscopy. The UV-vis spectra of GA, GA-CS and GA-g-CS are displayed in **Figure 2**. As can be seen, both GA-CS and GA-g-CS showed absorption peak between 250 and 350 nm. Meanwhile, in the spectra of GA-g-CS, the absorption peak at 262 nm shifted toward 272 nm compared to that of GA-CS. According to a previous study, GA exhibited characteristic absorption bands at 262 nm due to the π-system of the benzene ring (Xie et al., 2014). The red shift might be attributed to the decrease in energy required for the π–π^∗^ transition, because the covalent linkage of GA with CS brought the longer conjugated system. This result was also consistent with the information provided by FTIR analysis. The results above all indicated that GA was successfully grafted onto CS.

### Functional Properties of GA-g-CS Complexes

#### DPPH Radical Scavenging Assay

**FIGURE 3:**
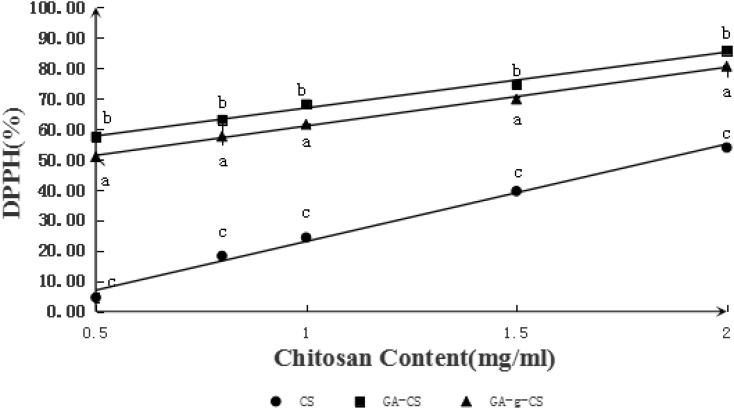
Scavenging activities on 2, 2-diphenyl-1-picrylhydrazyl (DPPH) radical of CS, GA-CS, and GA-g-CS. Data are presented as means ± SD of triplicates. Different letters at the same sampling time denote significant differences (*p* < 0.05) between treatments.

DPPH radical-scavenging activity is an index to speculate the resistance of samples under investigation to oxidation. Generally, the strong DPPH radical-scavenging activities can imply the high resistance of samples to oxidation. The DPPH radical-scavenging activities of all the samples are presented in **Figure 3**. It can be seen that the DPPH-scavenging activities of GA-CS and GA-g-CS increased with their concentrations. At the same time, the DPPH-scavenging activity of GA-g-CS was higher than that of unmodified CS. At a concentration of 2 mg/mL, the DPPH-scavenging activities of CS, GA-CS and GA-g-CS were 53.90 ± 0.16%, 85.85 ± 0.28%, and 80.60 ± 0.16%, respectively. The EC_50_ values of CS, GA-CS and GA-g-CS were 1.84, 0.08, and 0.43 mg/mL, respectively. Moreover, the antioxidant effect of chitosan after grafting gallic acid was significant by ANOVA. It is well-known that gallic acid is a potent antioxidant, which can scavenge free radicals and ROS (Hu and Luo, 2016). Thus, the antioxidant potential of CS can be improved after grafting with gallic acid. it could also attribute to the enhanced hydrogen and electron donating ability after conjugation of GA onto CS, which was gussed in previous studies. Xie et al. (2014), studied the application of complex (GA-g-CS) in lipid peroxidation and showed GA-g-CS exhibited greater antioxidant potential than native chitosan. They supposed that GA groups grafted on the structure of CS could attract more linoleic acid radicals by electrostatic affinity for GA-g-CS was easier to capture radicals than GA alone due to its enlarged macromolecular structure. Moreover, it was reported steric hindrance in phenolic hydroxyl groups by addition of methoxyl groups could enhance the antioxidant activity of CS (Ashrafi et al., 2017). Therefore, it was suggested that the conjugation of antioxidant phenolics onto chitosan was a useful approach to produce novel polymeric antioxidants.

#### Antibacterial Property

The common bacteria in pork can be divided into spoilage bacteria (*Pseudomonas, Acinetobacter, Brochothrix thermosphacta*) and pathogenic bacteria (*Escherichia coli, Staphylococcus aureus, Salmonella, Listeria monocytogenes*). The antibacterial properties of CS, GA-CS, GA-g-CS against *Pseudomonas, Acinetobacter, Brochothrix thermosphacta, Escherichia coli, Staphylococcus aureus, Salmonella*, and *Listeria monocytogenes* were studied. It can be seen from **Table 1** that the antibacterial capacity of chitosan after grafting gallic acid was significant. GA-g-CS had higher antibacterial capacity than unmodified CS. The antibacterial properties of GA-g-CS against *Pseudomonas, Acinetobacter, Brochothrix thermosphacta, Escherichia coli, Staphylococcus aureus, Salmonella*, and *Listeria monocytogenes* are 34.02 ± 0.05, 33.84 ± 0.09, 35.71 ± 0.17, 36.09 ± 0.12, 26.70 ± 0.20, 27.92 ± 0.01, and 21.20 ± 0.16 mm, respectively. The difference in antibacterial properties may be as a result of the differences in cell membrane structure and composition of each bacterial strain. It is well-known that CS is an effective antibacterial agent, since positively charged amino-groups on CS can interact with the negatively charged microbial cell components (Yang et al., 2016). The free amino groups on CS reacted with free hydroxyl groups on GA, thus forming macromolecular polymers with higher hydrophobicity. It was easier for GA-g-CS to react with the hydrophobic cell components and enhance the membrane permeability. Moreover as a natural polyphenols, the grafting of GA on CS can result in the enhancement of the antibacterial properties of GA-g-CS. Free amino groups of CS without the GA grafting, or the GA-g-GA may be the other reason for the improvement of antibacterial properties.

**Table 1:** The bacteriostasis ability of CS, GA-CS, and GA-g-CS.

	*Salmonella*	*Staphylococcus aureus*	*Listeria monocytogenes*	*Escherichia coil*	*Pseudomonas aeruginosa*	*Acinetobacter*	*Brochothrix thermosphacta*
CS	18.70 ± 0.30^c^	21.70 ± 0.20^c^	12.03 ± 0.08^c^	22.84 ± 0.44^c^	25.67 ± 0.12^c^	24.24 ± 0.13^c^	25.24 ± 0.21^c^
GA-CS	21.92 ± 0.03^b^	24.00 ± 0.35^b^	16.02 ± 0.04^b^	33.20 ± 0.19^b^	26.83 ± 0.07^b^	27.13 ± 0.27^b^	28.22 ± 0.16^b^
GA-g-CS	27.92 ± 0.01^a^	26.70 ± 0.20^a^	21.20 ± 0.16^a^	36.09 ± 0.12^a^	34.02 ± 0.05^a^	33.84 ± 0.09^a^	35.71 ± 0.17^a^

*The unit of the bacteriostasis circles’ diameter is mm. Data are represented by means ± SD (n = 3). Different letters (a, b, and c) at the same strain denote significant differences (p < 0.05) between treatments.*

### Application in Chilled Meat Preservation

#### pH

**FIGURE 4:**
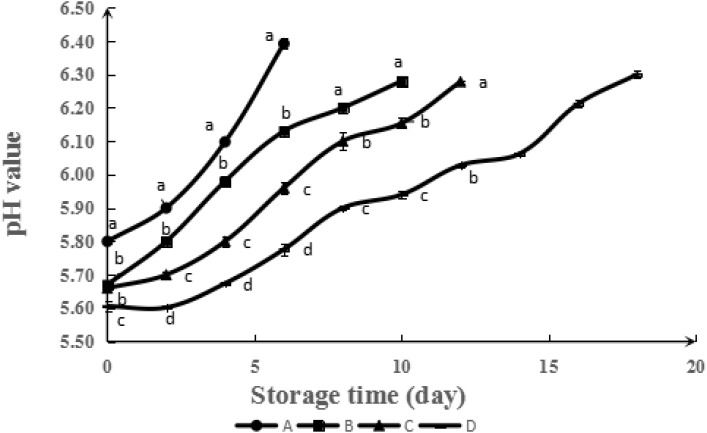
Evolution of pH value during simulated retail display (4°C) of pork untreated (A) and treated with CS (B), GA-CS (C), and GA-g-CS (D), respectively. Data are presented as means ± SD of triplicates. Different letters at the same sampling time denote significant differences (*p* < 0.05) between treatments.

CS, GA-CS, and GA-g-CS were applied for chilled meat preservation. The pH value can indirectly reflect the quality of meat. During storage, the formation of volatile gasses (e.g., trimethylamine and ammonia) produced by either microbial or endogenous enzymes can lead to the pH rising. According to **Figure 4**, the pH value of chilled meat increased gradually during storage. The initial pH value of chilled meat was about 5.8. With the prolongation of storage time, the pH values increased. According to the saliency analysis, the changes among control samples and samples treated by CS, GA-CS, and GA-g-CS were obvious on Day 6, Day 10, Day 12, and Day 18. According to the Chinese Standard (GB-T 9959. 2-2008) (2008), chilled meat gets spoiled at pH 6.4. Combined with **Figure 4**, it can be obtained the shelf of control samples and samples treated by CS, GA-CS and GA-g-CS were 6, 10, 12, and 18 days, respectively. GA-g-CS improved the shelf life of chilled meat obviously, which is potential for the preservation of chilled meat.

#### Thiobarbituric Acid Reactive Substances

**FIGURE 5:**
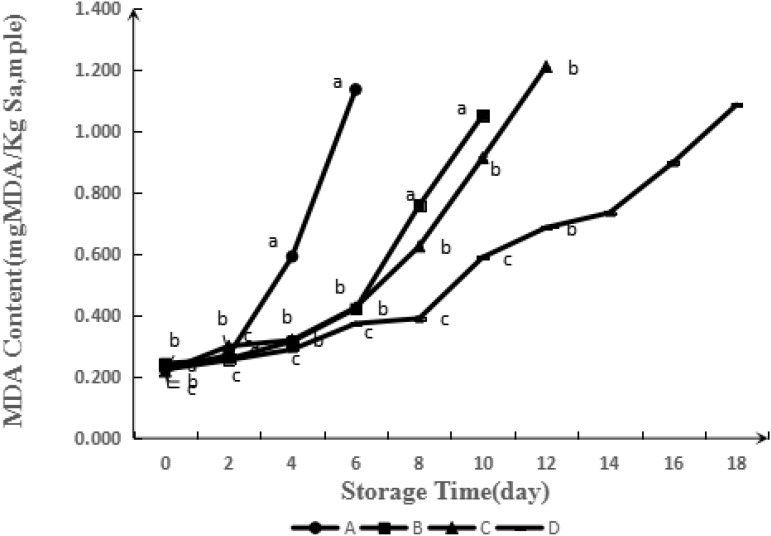
Evolution of TBARS during simulated retail display (4°C) of pork untreated (A) and treated with CS (B), GA-CS (C), and GA-g-CS (D), respectively. Data are presented as means ± SD of triplicates. Different letters at the same sampling time denote significant differences (*p* < 0.05) between treatments.

Lipid oxidation is normally found during the storage of meat products (Hansen et al., 2004). It is one of the important factors relating to the decline in meat quality, such as the formation of undesirable rancid flavor and poisoning. During the storage, free radicals in lipid oxidation were created by the attack of oxygen at the double bond in fatty acids (Kamaleldin et al., 2003). Generated primary lipid oxidation products, hydroperoxides, can lead to further oxidative polymerization to produce aldehyde, ketone, and alcohol compounds. TBARS is a common index for secondary lipid oxidation (Kamaleldin et al., 2003). **Figure 5** illustrates the influence of GA-g-CS on TBARS values of meat samples during refrigerated storage for 18 days. The TBARS value before storage was 0.20 mg MDA.Kg^−1^, which significantly increased with the storage time in all the samples. The changes among control samples and samples treated by CS, GA-CS, and GA-g-CS, analyzed by significance test, were obvious on Day 6, Day 10, Day 12, and Day 18, which was consistent with the results of pH value analysis. According to the Chinese Standard (GB-T 9959. 2-2008) (2008), the TBARS value above 1 mg MAD.kg^−1^ pork means the meat corrupted. The TBARS value of control sample increased to 1.14 ± 0.01 mg MDA. kg^−1^ on Day 6. On the other hand, the TBARS values of the samples coated with CS, GA-CS and GA-g-CS were lower than that of the control sample. The TBARS value was 1.05 ± 0.01 mg MDA.kg^−1^ for the samples coated with CS on Day 10, 1.21 ± 0.00 mg MDA.kg^−1^ for the samples coated with GA-CS on Day 12 and 1.08 ± 0.00 mg MDA.kg^−1^ sample for samples coated with GA-g-CS on Day 18. These values implied that the shelf life of control sample and samples coated with CS, GA-CS and GA-g-CS were 6, 10, 12, and 18 days, respectively. According to the previous reports, the interaction between amino groups and fat derivatives, as well as the chelation effect of chitosan with metal ions can inhibited lipid oxidation (Ashrafi et al., 2017).

#### TVB-N

**FIGURE 6:**
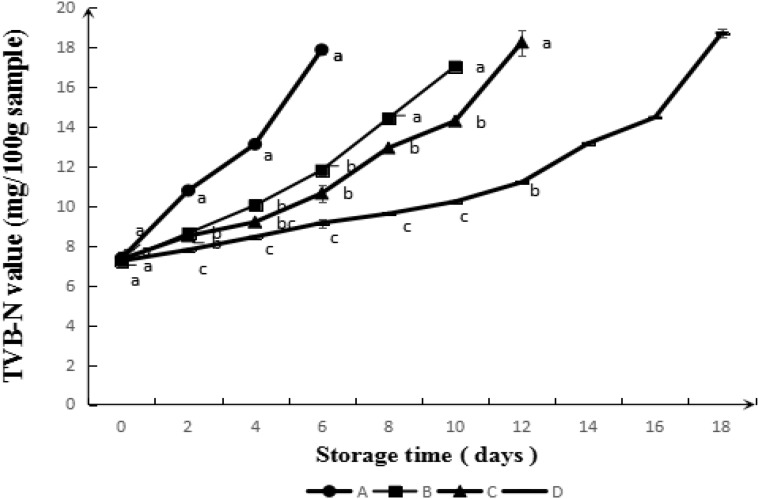
Evolution of TVB-N value during simulated retail display (4°C) of pork untreated (A) and treated with CS (B), GA-CS (C), and GA-g-CS (D), respectively. Data are presented as means ± SD of triplicates. Different letters at the same sampling time denote significant differences (*p* < 0.05) between treatments.

TVB-N is a index for freshness of regenerated meat. It can be seen from **Figure 6** that TVB-N value increased with storage time. The rising trend for samples treated by CS modified with GA by fmb-rL103 was smoother. According to the Chinese Standard (GB-T 9959. 2-2008) (2008), meat products got corrupted, when the TVB-N value reached 15 mg/100 g sample. In other words, it took 6 days for control sample to get putrid, whereas the samples coated with CS, GA-CS, and GA-g-CS kept fresh within the storage of 10, 12 and 18 days, respectively. This result was also in agreement with the results about TBARS. CS-g-GA was effective to extent the shelf life of the meat. It may be attributing to its higher antioxidant and antiseptic capacities. It has been reported that the lower bacteria number and higher antioxidant abilities were associated with the lower activity of amino acid decarboxylase, which contributed to the enhancement of TVB-N value (Wook et al., 2016).

#### Color

**FIGURE 7:**
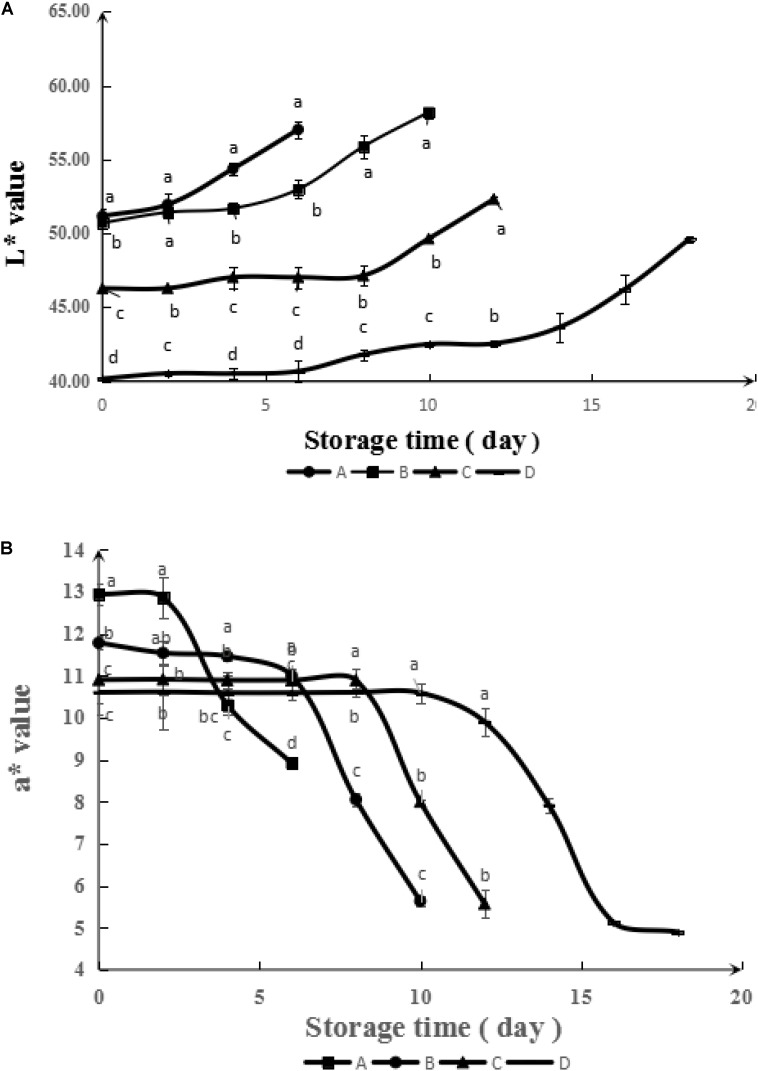
Evolution of color L^∗^ **(A)** and a^∗^ **(B)** during simulated retail display (4°C) of pork untreated (A) and treated with CS (B), GA-CS (C), and GA-g-CS (D), respectively. Data are presented as means ± SD of triplicates. Different letters at the same sampling time denote significant differences (*p* < 0.05) between treatments.

The purchasing desire of consumers largely depend on the color of food, since it is the most direct parameters reflecting the freshness of meat (Coombs et al., 2018). The evolutions of color parameters (*L*^∗^ and *a*^∗^) of meats during storage are illustrated in **Figure 7**. As can be seen, the *L*^∗^ and *a*^∗^ values of samples treated with GA-g-CS were lower than untreated samples due to the original color of GA-g-CS. Together with the extension of shelf life, the color of samples coated with GA-g-CS was also acceptable. During storage, the *L*^∗^ value for all the samples increased gradually, while the *a*^∗^ values declined. Meanwhile, the increasing rate of *L*^∗^ for samples coated with GA-g-CS was much lower than that of untreated sample. These changes indicated (Rodríguez-Carpena et al., 2012; Cando et al., 2014) the discoloration of refrigerated meat, which was usually attributed to the oxidation of oxymyoblogin into metmyoglobin. This protective effect of GA-g-CS was positively related with its antioxidant effect observed against TBARS formation. Thus, the relationship between lipid and myoglobin oxidation in red meat were established. Moreover, the water permeation from meat can also lead to the increase of *L*^∗^.

#### Microbial Assay

The environment in pork, including the chemical composition, water activity and pH can favor the microbial growth. Microbial level is an important index to assay the degree of meat spoilage. It can be seen from **Table 2** that, the bacteria showed an exponential growth during storage. The aerobic plate count (APC) of control samples was always higher than that of samples treated with CS, GA-CS, and GA-g-CS. The Chinese Standard (GB-T 9959. 2-2008) (2008) stated that the APC for meat products should stay below 10^6^cfu/g. It took 6 days for the APC of control sample to reach this threshold, while the APC of samples treated by CS, GA-CS, and GA-g-CS stayed within this range within storage of 10, 12, and 18 days, respectively. This result was coincident with the aforementioned indicators. CS grafted with GA was characterized by higher antioxidant and antiseptic properties. Thus, it was a more effective agent for the preservation of chilled meat.

**Table 2:** Evolution of the aerobic plate count (APC) and the coliform bacterial number (MPN) during simulated retail display (4°C) of pork.

	CK		CS		GA-CS		GA-g-CS	
	APC	MPN	APC	MPN	APC	MPN	APC	MPN
0	2.40 ± 0.00^a^	0^a^	2.56 ± 0.00^a^	0^a^	2.52 ± 0.00^a^	0^a^	2.62 ± 0.00^a^	0^a^
2	3.63 ± 0.22^a^	1.70 ± 0.22^a^	3.26 ± 0.00^a^	0^b^	3.18 ± 0.00^a^	0^b^	2.71 ± 0.00^b^	0^b^
4	5.26 ± 0.11^a^	3.26 ± 0.11^a^	4.04 ± 0.22^b^	1.92 ± 0.25^b^	3.82 ± 0.00^b^	0^b^	2.76 ± 0.00^b^	0^b^
6	6.34 ± 0.11^a^	5.46 ± 0.11^a^	4.82 ± 0.07^b^	3.00 ± 0.07^b^	4.61 ± 0.07^b^	1.78 ± 0.07^b^	3.09 ± 0.24^b^	1.57 ± 0.24^b^
8	–	–	5.28 ± 0.16^a^	3.99 ± 0.16^a^	5.08 ± 0.05^a^	2.86 ± 0.05^b^	3.40 ± 0.10^b^	1.78 ± 0.10^b^
10	–	–	6.08 ± 0.06^a^	5.08 ± 0.06^a^	5.51 ± 0.14^b^	4.18 ± 0.14^b^	3.71 ± 0.17^c^	2.20 ± 0.17^b^
12	–	–	–	–	6.18 ± 0.03^a^	5.26 ± 0.03^a^	4.87 ± 0.08^b^	2.91 ± 0.08^b^
14	–	–	–	–	–	–	5.26 ± 0.54	3.08 ± 0.54
16	–	–	–	–	–	–	5.84 ± 0.16	3.71 ± 0.16
18	–	–	–	–	–	–	6.26 ± 0.18	5.04 ± 0.18

*The unit of the aerobic plate count (APC) and the coliform group number (MPN) are log _10_ cfu/g. Data are represented by means ± SD (n = 3). Different letters (a, b, and c) at the same sampling time denote significant differences (p < 0.05) between treatments.*

According to **Table 2**, coliform bacteria also exhibited an exponential growth along with the increase of storage time. And differences in the number of coliform groups among control samples and samples treated by CS, GA-CS, and GA-g-CS were significant on Day 6, Day 10, Day 12, Day 18. Accordingly, the coliform bacterial number (MPN) was 5.46 ± 0.11 log_10_ cfu/g for controls samples on Day 6, 5.08 ± 0.06 log_10_ cfu/g for samples treated with CS on Day 10, 5.26 ± 0.03 log_10_ cfu/g for samples treated with GA-CS on Day 12 and 5.04 ± 0.18 log_10_ cfu/g for samples treated with GA-g-CS on Day 18. The aforementioned results indicated that the shelf life of control samples and samples treated with CS, GA-CS and GA-g-CS were 6, 10, 12, and 18 days, separately. The growth speed of coliform bacteria in samples treated with GA-g-CS was lower than that of control samples, demonstrating that GA-g-CS was effective for the inhibition of microorganisms on meat.

## Conclusion

In the study, CS successfully grafted with GA by fmb-rL103. The UV-vis and FT-IR analysis confirmed CS grafted with GA through covalent bond, such as ester bonds and amide group. After grafting with GA, CS had a higher antioxidant and antiseptic capacities than the CS along. Furthermore, coating chilled meat surface with GA-g-CS solution lead to a significant extension of their shelf life in term of appearance, oxidation stability and microbiological stability during 18 days of chilled storage. The shelf life was extended 6–18 days after GA-g-CS treatment. Therefore, fmb-rL103 is a good catalyst for CS to graft with GA. GA-g-CS has promising applications in meat preservation.

## Author Contributions

CZ designed the research, analyzed the data, and wrote the paper. MZ performed the research, analyzed the data, and wrote the paper. YZ performed the research. ZL, HZ, XB, and FL analyzed the data.

## Conflict of Interest Statement

The authors declare that the research was conducted in the absence of any commercial or financial relationships that could be construed as a potential conflict of interest.
